# Meetings of Societies

**Published:** 1913-03

**Authors:** 


### MEETINGS OF SOCIETIES.

### Bristol Medico-Chirurgical Society.

*December* 11*th*, 1912.

Dr. Walter C. Swayne, President, in the Chair.

The following demonstrations of clinical methods were given and specimens shown :—**Bronchoscopy and Œsophagoscopy,** by Dr. Watson-Williams and Mr. A. J. Wright ; **Intravenous Ether Anæsthesia,** by Mr. A. L. Flemming ; **Intestinal Skiagraphy with Bismuth Meal,** by Mr. F. G. Bergin ; **Gastroscopy,** by Dr. Stack ; **The Polariscope,** by Dr. J. A. Nixon ; **Naso-Pharyngoscopy,** by Dr. Watson-Williams and Mr. A. J. Wright ; **Carbon Dioxide Estimation in the Blood** and **Solid Carbon Dioxide Treatment of Nævi,** by Dr. A. Rendle Short ; **Urinary Acidosis estimation,** by Dr. R. S. S. Statham ; **Skiagrams,** by Dr. J. Taylor ; **Pathological Specimens,** by Professor Walker Hall, Dr. Scott Williamson, Dr. Kay-Mouat and others.

*January* 8*th*, 1913.

Dr. Walter C. Swayne, President, in the Chair.

Dr. J. A. Nixon gave a demonstration on the technique of the **Intravenous administration of neo-salvarsan.** (See p. 24.)

Dr. C. W. J. Brasher, in a paper on **Intermittent swelling of the parotid gland,** related the case of an adult female who had had attacks of acute parotid swelling at intervals for more than ten years. A vaccine of the *Micrococcus catarrhalis* grown from the interior of Stenson's duct gave a year of freedom from attacks, but the condition had since recurred.—Dr. J. O. Symes said that he had seen a boy with bilateral parotid swelling occurring every evening for some weeks following an attack of mumps, and Dr. Carey Coombs said that he had seen a similar condition affecting the submaxillary glands.

Dr. R. S. S. Statham showed a specimen of primary **Rupture of the pregnant uterus before labour.** When the patient, who was eight months pregnant, was first seen it was thought to be a case of concealed accidental hemorrhage, but laparotomy revealed an extensive rupture of the uterus and the child free in the peritoneal cavity. She had been curetted a year previously for a septic abortion, and no other cause could be found.

Dr. F. H. Edgeworth related a case in which **Phosphaturia and oxaluria** alternated. (See p. 18.)—Dr. Brasher mentioned a case of oxaluria accompanied by hæmaturia and renal colic. (See p. 21.)

---

*February 12th*, 1913.

Dr. Walter C. Swayne, President, in the Chair.

The meeting was devoted to a discussion on the **Treatment of chronic constipation.** The discussion was opened by Dr. F. H. Edgeworth (see p. 40) and Mr. T. Carwardine.

J. Lacy Firth.

A. J. Wright, *Hon. Sec.*

